# The Problem of Selecting the Parameters of the Numerical Model of the Heating Process with a Point Heat Source and Its Experimental Verification

**DOI:** 10.3390/ma16020532

**Published:** 2023-01-05

**Authors:** Michał Sobiepański, Joanna Wróbel, Adam Kulawik

**Affiliations:** 1 Department of Mechanical Technology, Czestochowa University of Technology, Al. Armii Krajowej 21, 42-201 Czestochowa, Poland; 2 Department of Computer Science, Czestochowa University of Technology, Dabrowskiego 73, 42-201 Czestochowa, Poland

**Keywords:** TIG, heat process, numerical model, parameters of boundary conditions, experimental research

## Abstract

The paper presents an analysis of the problem of selecting the parameters of the model describing the heating process. Heating is treated as a part of the process of heat treatment of elements such as axles and shafts using a heat source in the form of an electric arc. For this purpose, an experimental stand was made and research was carried out to analyse the temperature in the control node. Cylindrical specimens with a constant cross-section made of medium carbon steel AISI 1045 were used as the test objects. A device using TIG technology was used as the heat source. Due to the heating of the element—its rotational movement—it was necessary to use a non-contact measuring device. The construction of the research stand is a representation of the developed industrial stand. In addition, calibrations of the non-contact measuring system were performed using a thermocouple system. Comparing the results obtained from the experiment and the numerical model showed a fairly high convergence of the adopted numerical parameters (the difference between the experiment and the numerical model did not exceed 6.5%). In addition, an analysis of the surface of the samples was performed for the occurrence of remelting by determining its roughness and waviness.

## 1. Introduction

Modern industry is increasingly characterised by small batch or even unit production. This area is also affected mainly by surface improvement processes such as heat, chemical or mechanical treatment. Furthermore, taking into account the contemporary problems strongly affecting the energy-intensive industry, the application on a larger scale is accurate thermal processing carried out with a moving heat source. In this scope, heat sources such as induction heating, treatment with a laser source, gas source or electric arc are mainly used [1,2,3].

Heat treatment with a moving heat source is characterised by the need to establish the exact path of the heat source transition to reflect volumetric processing without clear boundaries at the edges of the source interaction zone. Path planning strategies are related to establishing new surface properties of steel parts (hardness, phase composition, chemical composition) uniformly both on the surface and inside the material [4,5]. Due to their characteristics, point heat sources can also be used to process large elements. As a modern area of development of this technology can be mentioned work related to the welding of underwater structures. Elements of this type must, for technological reasons, be locally heated (preheating or tempering) using, for example, an induction source [6,7].

Due to corrosion processes during high-temperature processing, it is essential to consider the effect of shielding gases not only on the chemical properties of the surface [8], but also on its geometric parameters [9]. Among the high-temperature heat sources, the processes associated with the Tungsten Inert Gas (TIG) method and its wide range of modifications occupy an important place [10]. The TIG method is used in research work and described in the vast majority of cases with remelting, often assisted by additional substances or modifications to the method and considered due to the analysis of the behaviour of the remelted area [11].

An essential element of the simulation process is its possibly accurate experimental verification. In the case of heat treatment with TIG, an important parameter is temperature recording, one of the available measurement methods [12]. The measurement of moving parts excludes the use of touch methods. It is necessary to use non-contact methods with accurate determination of measurement parameters for the specific measured surface [13]. It is important to study the effectiveness of simulation in predicting heat source parameters due to the large range of variability of welding conditions [14].

Taking into account the cost-intensity of the devices in the process in question, their efficiency and the small depth of fusion, a heat treatment station using an electric arc obtained with a non-consumable electrode in a noble gas shield (TIG) [15] was chosen for the presentation of the method. Such a choice of tool allows hardening without degeneration of the near-surface area at the point of interaction of the electric arc. The selection of the power of the device operating especially on the material at the surface will affect not only the hardness of the surface but also the possible changes in the geometry associated with local remelting, the boundaries of the heat affected zone or phase transformations in the solid state.

In the papers on the heat treatment process, both model simulation and correctly performed experimental research are important. This paper aims to verify the possibility of modelling the modern process of manufacturing axles and shafts of medium-carbon steel used for heating by an unusual heat source, which is the electric arc of a TIG welder. It is assumed that, through such analysis, it will be possible to determine the parameters of the above process for axles and shafts with variable cross-sections but not significantly different from the tested diameters. The results of experimental tests performed for a particular family of geometries (ranges of diameters, lengths or materials) often do not allow us to determine the technological parameters for other steel parts. For this reason, an experiment should be performed to verify the numerical model, which can then be easily transferred to modelling a wide range of steel parts. The geometries of the analysed parts were chosen because of the frequency of axisymmetric elements with variable diameters that are heat-treated, for which it is complicated to perform uniform treatment with an induction source. Thus, the selection of tools and methods, as well as the research area, refers to an energy-efficient process performed on inexpensive equipment for low-volume production. The use of higher power sources, where micro-remelting of the surface layer occurs, can also be used in the field of surface improvement of additive materials. However, this paper focuses only on thermal phenomena.

Research in the field of model calibration describing the heating or cooling process is not new. Quite often, the selection of model parameters is performed based on an experiment, also in complex multi-scale models or using modelling for several spatial systems [16,17]. However, according to the authors, the analysed technique of heat treatment of axisymmetric elements often used in the automotive or engineering industry has not been analysed this way. The experiments performed for the paper concern a specific hardening technology for steel components. The selection of the shape of the samples and the parameters of the experiment itself should be understood in this context. Thus, the paper does not refer to the welding process, in which the most important element is the melting of the material. In the modelled process, remelting is a messy phenomenon (hence the performance of additional tests of roughness and waviness of samples). Determining the parameters of devices using an electric arc such as TIG in the temperature range without remelting is rarely found in the literature.

The paper attempts to carry out numerical analysis and experimental heat treatment in the area of heating for solid axisymmetric elements with a constant cross-section made of medium carbon steel (AISI 1045). For this purpose, an experimental stand allowing TIG heating of samples with a constant cross-section up to 1 m length with the possibility of setting any circular or spiral path of the heat source passage was made. Based on the experiment, the model was calibrated in the authors’ own application based on the finite element method (FEM) in the field of temperature modelling.

## 2. Experiment

**Figure 1:**
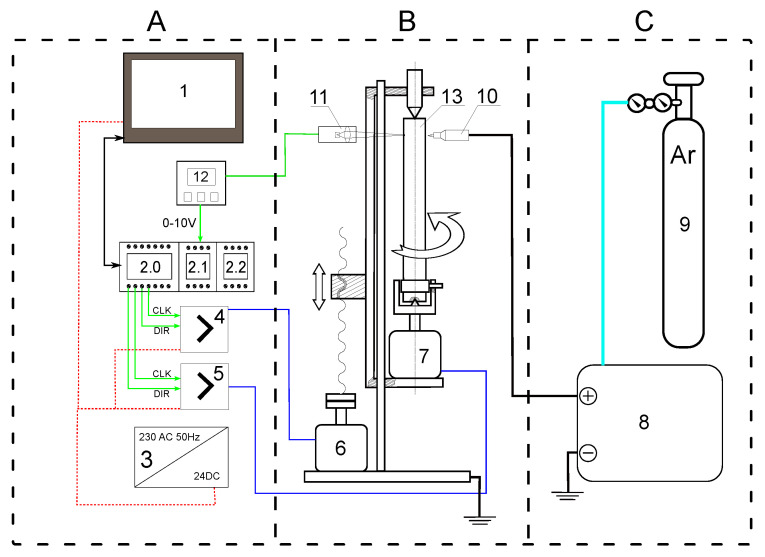
The experimental stand structure: 1, operational panel; 2.0, PLC main unit; 2.1, analog to digital module; 2.2, thermocouple module; 3, power supply; 4, 5, stepper motor drivers; 6, 7, stepper motors; 8, current source; 9, compressed argonium tank with pressure reduction unit; 10, welding grip with tungsten electrode; 11, measuring head of laser pirometer; 12, measuring transducer/amplifier of laser pirometer; 13, analysed sample.

The selection of the parameters of the numerical model used for the in-depth analysis and the number of possible cases occurring in the manufacturing process was made based on experimental research. A stand was used for heating steel bars with the TIG method, with the possibility of stationary heating and in a circular or spiral path. A research stand consisting of three main blocks was built: control and measuring system (A), mechanical part (B), and current source with argon reservoir (C) (Figure 1).

Heating was carried out using an invention welder KEMPPI MasterTig 235ACDC (element 8, Figure 1). The welding current and the welding voltage were acquired directly from the welding machine LCD display. Both parameters are key parameters of the process, thus they are controlled by the microprocessor of the welding machine and are stable during the experiment. The parameters of the welding machine allowed us to maintain the set distance of the electrode from the surface of the sample throughout the heating process. The heat supply was started automatically by the PLC controller bypassing the switch located on the welding gun.

**Figure 2:**
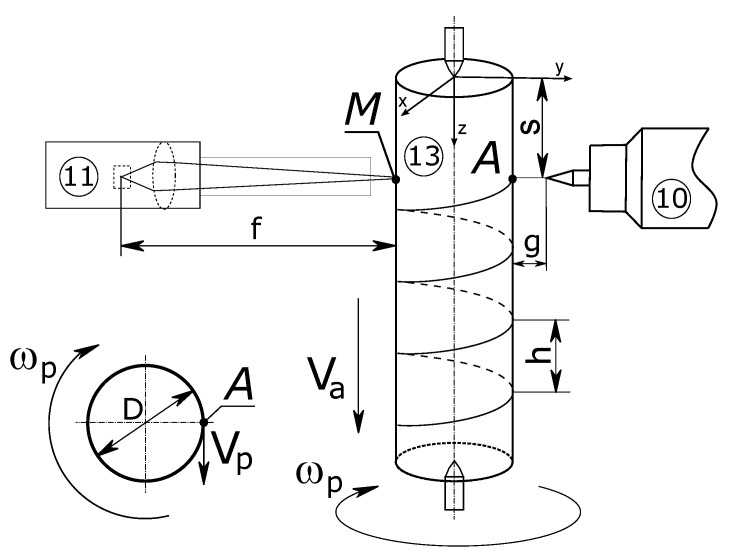
Scheme of the analysed task: 10, heat source; 11, measuring system; 13, analysed sample.

Steel specimens with a length of 0.2 m were used for the experiment (Figure 2). The specimen was placed in the work area of the workstation, which allowed rotational and feed movements of the test element. The movement of the sample was forced by the stationary position of the heat source. This enabled the area of influence of the electric arc to move along the helix line. The movement path of the heat source was described by the following parameters: axial rate ($V_{p}$) and rotational rate ($\omega_{p}$). Thus, the distance between successive lines of the helix depends on the linear velocity, while the rotation rate of the sample depends on $\omega_{p}$. For technological reasons, the non-contact temperature measurement point, in order to eliminate the influence of the electric arc on the measurement error, was placed at the end of the diameter coming out of the sample heating point (*A*).

The temperature measurement system consisted of a RAYTEK MI3 pyrometer equipped with a measuring head MI31002MSF1 (element 11, 12, Figure 1) [18]. The control system of the experimental stand was based on the PLC controller and HMI panel. The PLC controller consists of a main unit FATEK FBs-24MCJ2-D24 (element 2, Figure 1) equipped additionally with two expansion units: a six channel analog to digital converter (FATEK Fbs-6AD) and six channel thermocouple module (FATmodelling controller operational panel (WEINTEK MT8102iP)) were used as the interface necessary for supervising of the experiment and as the recorder for acquired data (element 1, Figure 1). The control system based on PLC controller has some features, which are helpful during experiments: high resistance to electrical disturbances, easy parametrization by human machine interface, built-in ready to use functions for numerical control of drives, built-in analog to digital converter.

Due to the high variability of the material of the part (especially it’s surface), an accurate selection of the emissivity coefficient based on the tables is practically impossible [19]. Considering that the thermocouple measurement of a rapidly rotating part is physically impossible, this measurement should be performed using a non-contact method. For the above reasons, it is necessary to calibrate the emissivity coefficient set for the laser pyrometer with the thermocouple measurement for the sample material. Stationary measurement (without movement) will allow us to determine the correct parameters and will also allow us to check the linearity of the characteristics of the device for the non-contact measurement device. The experiment was divided into two parts (I and II). The first part concerned calibration, while the second part involved tests related to the correctness of the heating model along the spiral path for different technological parameters. The following common conditions of the experiment were established:

- Sample material: steel ANSI 1045;
- Sample diameter: 0.012 m;
- Electrode-sample distance: 0.001 m;
- Electrode angle: 30°;
- Focal length: f = 0.2 m;
- Shielding gas: argon;
- Measurement zone diameter: 0.002 m;
- Cooling in air to ambient temperature.

Conditions of the calibration experiment:

- Sample length: 0.105 m;
- Location of the heat source in the middle of the sample height, s = 0.05025 m;
- Heat source parameters: current 60 A, voltage 9.8 V;
- TIG heating time: 280 s;
- Stationary sample: $V_{a}$, $V_{p}$ = 0.

**Figure 3:**
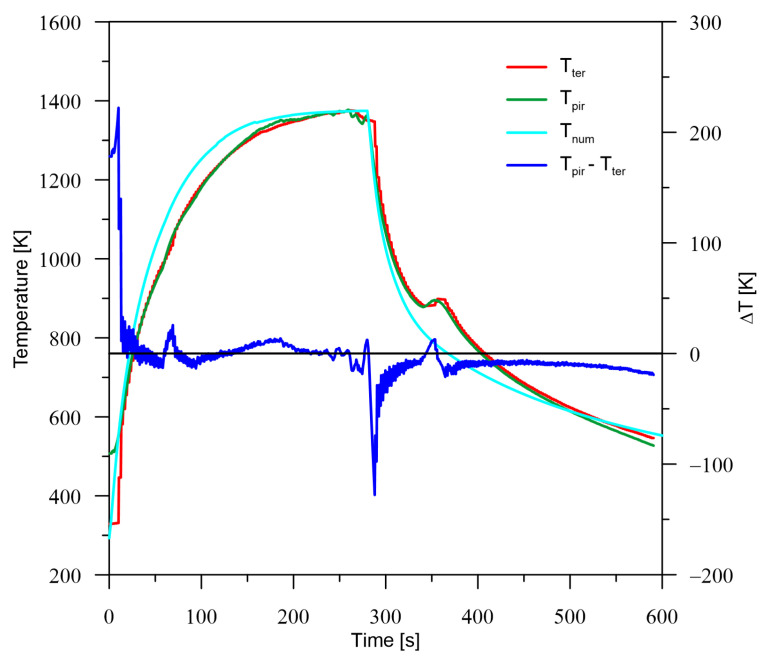
Temperature distribution at the measuring point (*M*) for: thermocouple ($T_{ter}$), pyrometer ($T_{pir}$), numerical model ($T_{num}$).

Calibrations were carried out for a rod of the same diameter as the samples used in the experiment. It was assumed that the length of the bar should be at least eight times the diameter of the rod. Therefore, a height of 0.105 m was assumed. This dimension resulted from numerical analyses for different specimen lengths and the proportion of the impact magnitude of the direct heat source to the analysed specimen diameter and length. The adopted length allows for stability in the range of maximum heat treatment temperature without the need to suddenly start cooling after the heat source passes outside the sample area. It is assumed that the heat treatment process in the manufacturing process is subjected to axes and shafts with variable cross-sections, and this treatment must have stable parameters. To reduce the effect of fixation, it was assumed that the heat source would be placed in the middle of the specimen height. Due to the simultaneous verification of the pyrometric device by the thermocouple system, the rotation of the sample was abandoned. To ensure accurate measurements for the pyrometer, a measuring path shield in the form of a metal tube with the diameter of the end of the pyrometric system was used. The end of the heating period was set for the moment when the derivative of the maximum temperature determined for subsequent measurements was close to 0 (calculation time 280 s). The sample was then allowed to cool freely with air by interrupting only the heating process. The purpose of this test was to determine the emissivity value of the tested material (increasing the accuracy of pyrometer measurement). Analysing the obtained results (Figure 3), it can be noticed that there is a high convergence of results from the thermocouple and pyrometer system if the emissivity coefficient is set at $\xi_{pir}$ = 0.8.

In Figure 3, it can be seen that the large error at the beginning of the measurement is related to the range of the pyrometer device (from 523 K). The second area of increased error (increased difference between temperatures) is the cooling start time is the result of high cooling rates.

The calibration process was also used for the initial verification of heat source parameters such as efficiency, power and radius, as well as the method adopted to include cooling. It can be seen in Figure 3, that the combination of cooling and simultaneous heating is quite consistent with experimental measurements. However, there is a significant difference in the area of cooling itself. Due to the fact that the work focuses on the time when the heat source is turned on, the errors of the cooling process were omitted from further analysis.

Experimental conditions of heating along a spiral path:

- Sample length: 0.2 m;
- Starting position of the heat source at the middle of the sample height, s = 0.02 m;
- Heat source parameters:
  − Current: 40 A, voltage 9.5 V;
  − Current: 60 A, voltage 9.8 V;
  − Current: 80 A, voltage 10.7 V.
- TIG heating time: 180 s;
- Axial rate of the heat source: $V_{a}$ = 0.001 m/s;
- Specimen rotation rate:
  − $\omega_{p}$ = 1 rot/s, ($V_{p}$ = 0.03767 m/s);
  − $\omega_{p}$ = 2 rot/s, ($V_{p}$ = 0.07534 m/s).

It was assumed that the calibration of the model parameters would be carried out for the heat treatment process of an axisymmetric element using a point source moving along a spiral path. It was also presumed that the parameters set during the calibration process will not change. For the above assumptions, the obtained results were compared for three different current parameters of the heat source and two rotational rates, while maintaining one axial speed.

## 3. Determination of Parameters of the Numerical Model Based on the Experiment

The paper analyses the experiment in the field of thermal phenomena. The process of heating and cooling for the selected geometry and boundary and initial conditions determines the modelling in full 3D space. Therefore, the basis of the modelling process is the numerical solution of the heat transfer equation with volumetric sources:

(1) $$\nabla \cdot \left(\lambda \nabla T\right)-\rho c\frac{\partial T}{\partial t}=Q_{v}$$

where *T* (K) is the temperature, *t* (s) the time, λ (W/mK) is the thermal conductivity, ρ (kg/m${}^{3}$) is the mass density, *c* (J/kg K) is the effective thermal capacity, $Q_{v}$ (W/m${}^{3}$) is the volumetric heat source.

The heat transfer equation was solved using the finite element method with Euler coordinates. A mesh was made using cubic elements with trilinear approximation compacted at the edge. Due to the use of Euler coordinates, the boundary condition had to be implemented on a uniformly compacted mesh along the length of the element. The selection of spatial discretization was done by performing test computations for a series of rod cross-section meshes (2D task). This provided the possible maximum size of boundary elements, the size of which does not significantly affect the level of calculation accuracy. Based on the boundary discretization in the cross-section, an analysis of the results was also carried out for a 3D grid limited to half the length of the target sample (0.105 m). On the boundary surface, boundary elements in the shape of quadrangular elements were adopted. It was assumed that the dimension of the boundary element in the direction of the rod length would be identical to the dimension in the direction along the perimeter of the cross-section. This ensured correct modeling of the heat source on the 3D surface and appropriate discretization of the boundary area due to the pitch of the helical transition path of the heat source. In contrast to the paper [18], the displacement of the heat source both radially and axially was done in every time step and was, respectively, 30 displacements per rotation (1 rot/s) and 15 displacements per rotation (2 rot/s).

All calculations for both 2D and 3D space were carried out in software developed by the authors and implemented in C++ using the Intel^®^ oneAPI Math Kernel Library for arithmetic calculations.

Based on the authors’ previous paper [18], it was determined that the efficiency of the source used should be around 70%. The voltage value was taken from measurements obtained in the experiment. For modelling, the surface heat sources with a boundary condition of type II described by Teixeira et. al. [20] and Weman [15], whose equations are as follows:

(2) $$\begin{array}{c} q=\frac{Q}{2\pi R^{2}}exp\left(-\frac{{\left(x-x_{0}\right)}^{2}+{\left(z-z_{0}\right)}^{2}}{2R^{2}}\right)\\ Q=UI\xi \end{array}$$

where *Q* (W) is the power of the heat source, *R* (m) is the radius of the source, $x_{0}$, $z_{0}$ are the coordinates of the centre of the heat source (m), *U* (V) is the arc voltage, *I* (A) is the arc current, ξ is the arc efficiency.

Cooling was performed with a boundary condition of type III:

(3) $$-\lambda \frac{\partial T}{\partial n}\left(x_{\alpha },t\right)=\gamma \alpha_{air}\left(x_{\alpha },t\right)\left(T\left(x_{\alpha },t\right)-T_{\infty }\left(x_{\alpha },t\right)\right)$$

(4) $$\alpha_{air}=\left\{\begin{array}{cc} 0.0668\times T & T_{0}<T<773\;K\\ 0.231\times T-82.1 & T\geq 773\;K \end{array}\right.$$

where γ is an additional coefficient, $\alpha_{air}\left(W/\left(m^{2}K\right)\right)$ is the heat transfer coefficient, $T_{\infty }$ (K) is the ambient temperature (K).

The value of the heat transfer coefficient for air defined by intervals was adopted according to the article [21]. Although all the conditions of the simulation were met, this did not allow us to obtain convergent results. Probably, this was influenced by the method of attachment, ambient conditions, as well as the geometry of the object. For this reason, an additional coefficient γ was introduced and determined for a stationary specimen at the level of γ = 1.15. The area of influence of the cooling condition was assumed to apply to the entire geometry of the specimen except for the area of influence of the heat source. Cooling was also assumed during the operation of the heat source.

The element discretisation is constant for each cross-section of the geometry. The mesh consists of cubic elements, which form a structural mesh (rectangles) on the boundary surface. Following the axis of symmetry, it was established that the number of elements would be 200, which results in the number of spatial elements in the mesh at the level of 412,800 (452,729 nodes). The compaction at the boundary layer was intended to account for possible remelting (which did not occur) and to account for differences in rates when determining phase transformations in the solid state. However, due to the material and cooling medium, only a ferritic–pearlitic structure was formed during cooling.

Parameters of the numerical model:

- Simulation parameters according to the experimental conditions;
- Initial temperature: 293 K;
- Material properties as for AISI 1045 steel [22];
- Time step:
  − Calibration process: 2 s;
  − Heat treatment process: 1/30 s;
- Consideration of cooling condition III type as for air on all boundaries, excluding the area of influence of the heat source;
- Heat source implemented with condition II type—consideration only of surface heat source (Table 1);
- Power of the heat source calculated from the data of from experiment, efficiency coefficient of the source equal 0.7;
- Mesh parameters:
  − At the outer boundary, the average dimension of the finite element was 0.0004 m;
  − At a depth of 0.001 m, it increased to a level of 0.002 m to reach a size of 0.01 m in the middle of the element.

## 4. Results

**Figure 4:**
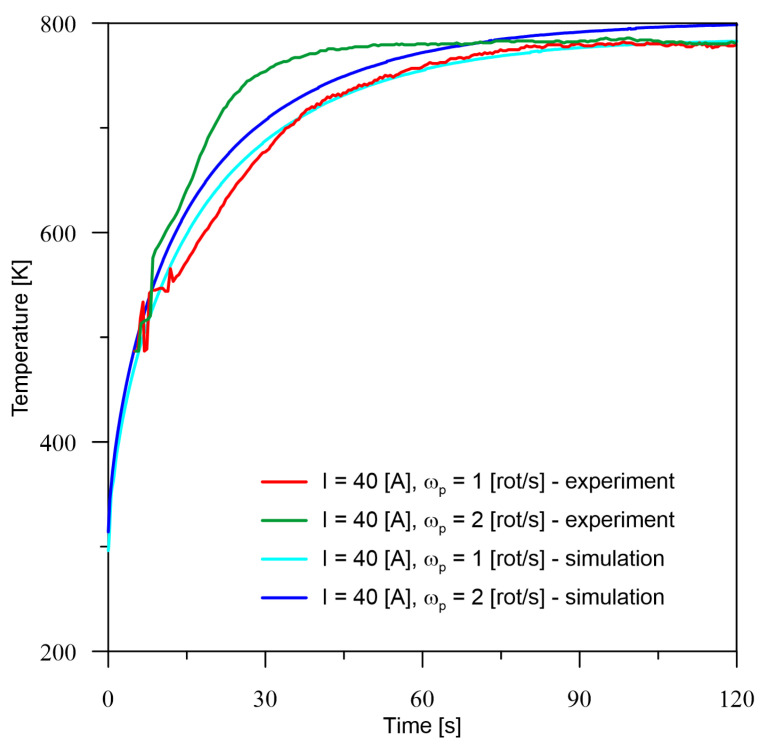
Comparison of the results of the experiment and the numerical model for *I* = 40 A.

**Figure 5:**
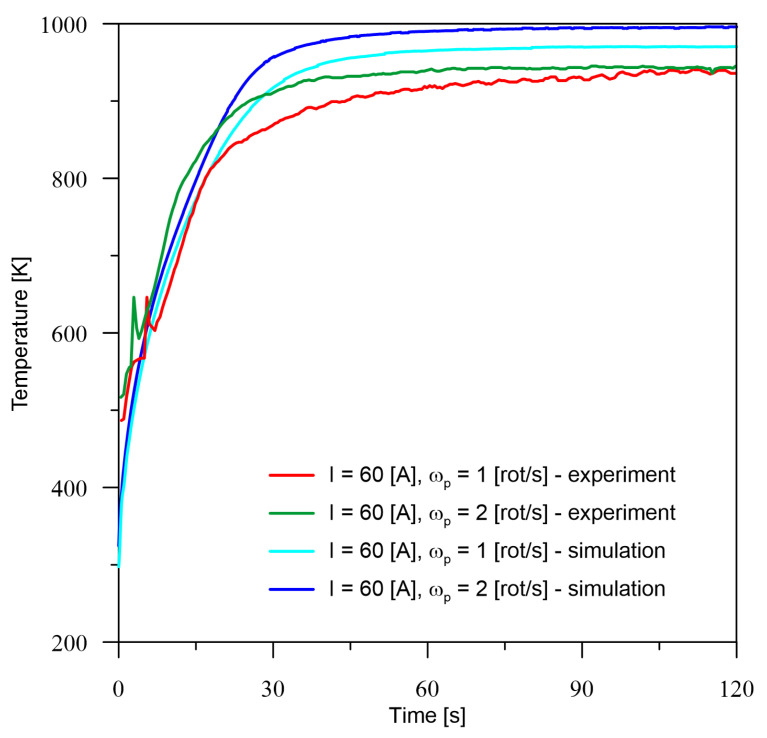
Comparison of the results of the experiment and the numerical model for *I* = 60 A.

**Figure 6:**
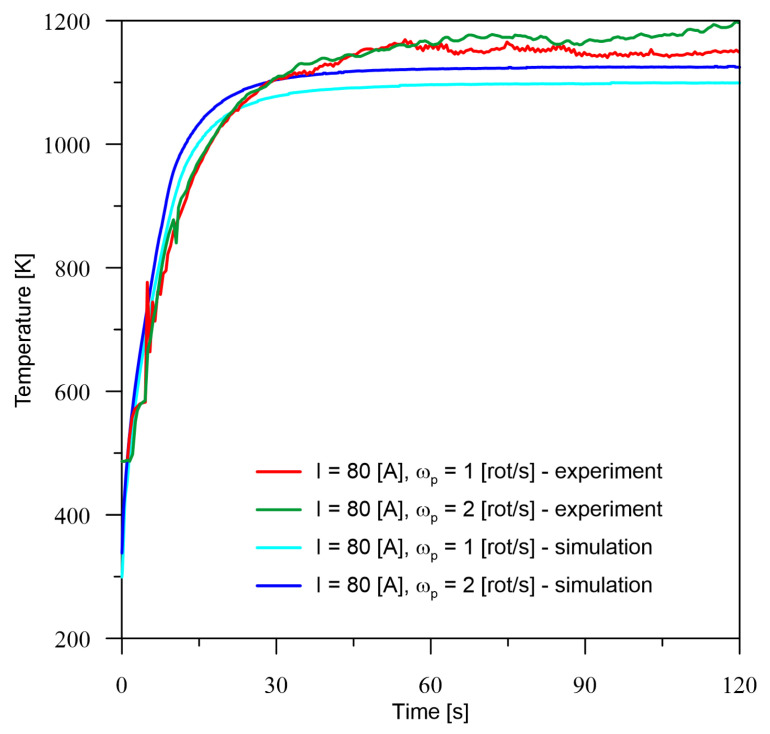
Comparison of the results of the experiment and the numerical model for *I* = 80 A.

According to the presented experimental and simulation conditions, verification measurements were made at point *M* (Figure 2). The temperature registered at point *M* for all six cases of current parameters of the heat source (Table 1) is quite consistent with the experimental results. The tendency to increase the temperature with an increase in the number of rotations can be observed. Probably, this is also associated with a decrease in the maximum temperature, which is confirmed by the surface profiles included in the section number 5. Analysing the obtained temperature values from both experiment and simulation, there is no trend in the underestimation or overestimation of values by the model, because for 40 A (Figure 4) there is a minimal overestimation, for 60 A (Figure 5), there is an overestimation of values at a higher level, while for 80 A (Figure 6), we notice an underestimation of the values.

Based on the differences between the obtained values from the numerical model and experiment presented in Table 2, it can be seen that the percentage error is no greater than 6.5%. For the smallest analysed current value, the larger error occurs in the zone before temperature stabilisation and drop to about 1% after the process stabilises. For the current of 80 A, overestimation can be observed for both cases at the initial time and stabilisation of the error at a level of up to 5% in the area of constant thermal conditions. On the other hand, for a current equal to 60 A, it can only be seen that the error is more or less at a constant level in the area of process stabilisation and this is due to the overestimation of temperature values by the numerical model.

**Figure 7:**
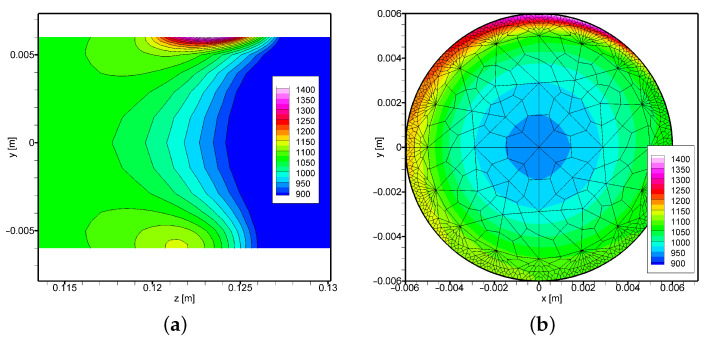
Temperature field in the cross-section: (**a**) longitudinal (x = 0 m), (**b**) transverse (z = 0.1235 m), in the area of maximum temperature (*t* = 100 s, *I* = 80 A, $\omega_{p}$ = 1 rot/s).

**Figure 8:**
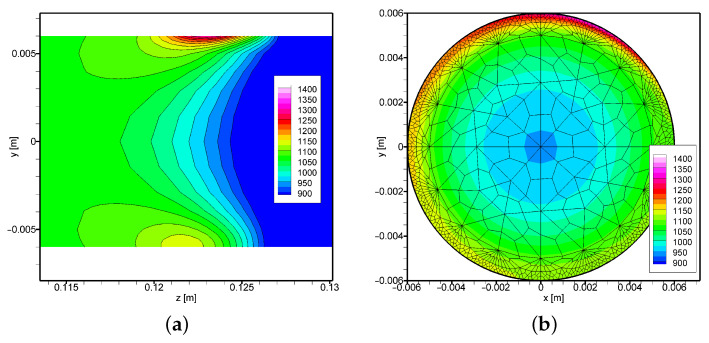
Temperature field in the cross-section: (**a**) longitudinal (x = 0 m), (**b**) transverse (z = 0.1235 m), in the area of maximum temperature (*t* = 100 s, *I* = 80 A, $\omega_{p}$ = 2 rot/s).

**Figure 9:**
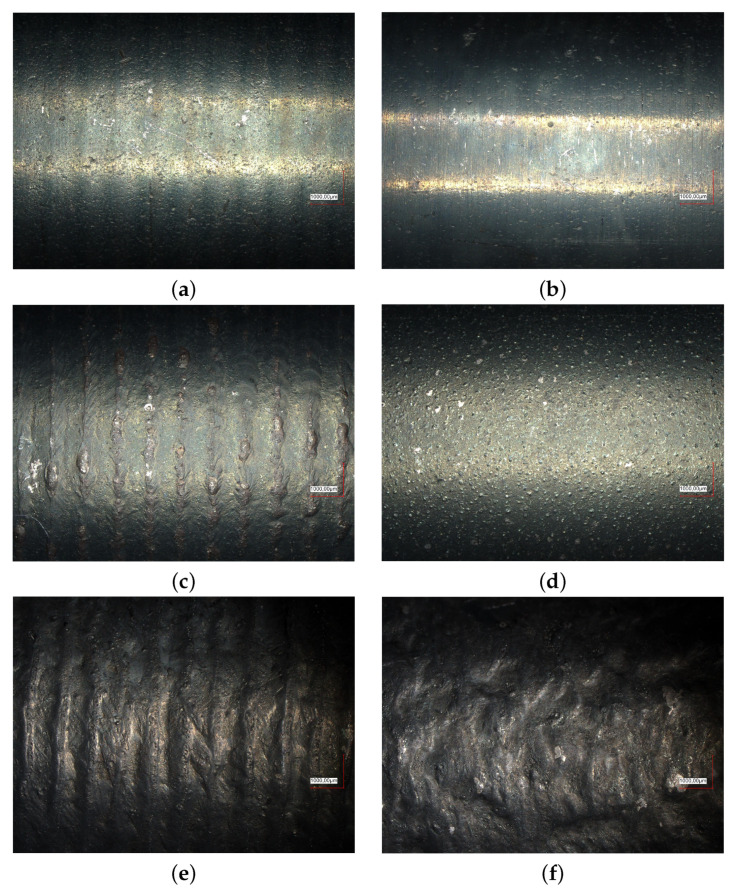
Surface condition of the sample after heat treatment experiment: (**a**) *I* = 40 A, $\omega_{p}$ = 1 rot/s, (**b**) *I* = 40 A, $\omega_{p}$ = 2 rot/s, (**c**) *I* = 60 A, $\omega_{p}$ = 1 rot/s, (**d**) *I* = 60 A, $\omega_{p}$ = 2 rot/s, (**e**) *I* = 80 A, $\omega_{p}$ = 1 rot/s, (**f**) *I* = 80 A, $\omega_{p}$ = 2 rot/s.

The experimental conditions allowed verification of the numerical model at the measurement point *M* (Figure 2). Based on the obtained accuracy, it can be assumed that the temperature distribution determined from the numerical model is correct. Therefore, the paper also presents the results from the numerical model in two significant cross-sections. The presented results (Figure 7 and Figure 8) refer to longitudinal and radial cross-sections containing the area of occurrence of the maximum temperature value. As expected, for the case when the rotational rate is 2 rot/s (Figure 8), we have a greater temperature equalisation both analysing the external area and the internal area of the cross-section (Figure 8 and Figure 9). The difference between the values measured at point *M* is only 20 K. In comparison, the maximum temperature value at point *A* differs by more than 70 K. A more even distribution also has a positive effect on the condition of the surface (see Figure 9a–d). It can be noted that the analysed temperature distribution will always contain large temperature gradients considering the area of direct influence of the heat source. However, the differences after half the rotation with respect to the center of the rot are not so significant and amount to about 100 K. Analysing the temperature distribution, it can also be concluded that it will be problematic to maintain thermal conditions at the end of the process in the area when the axisymmetric element ends. In this case, we will have to deal with the heated element on one side and the boundary of the element on the other side, so there is certainly a rapid increase in temperature in this area.

The discussed type of treatment is not suitable for practical use for rods of small cross-sections. A cross-section large enough is required to ensure that the heat absorption through the interior of the element will be provided at a relatively uniform level along the entire length of the axisymmetric element. Heat treatment (through-hardening) would only be possible if the value of the heat source power could be gradually reduced as the element heated up and the heat dissipation conditions changed.

## 5. Additional Elements of the Experiment

In addition, results on surface quality after the heat treatment process were obtained from the experiment. Although they do not directly relate to the numerical model, they can be used in the future to verify and improve the heat source model. Although there is a fairly large convergence between the temperature measured opposite the heat source location (Figure 4, Figure 5 and Figure 6), the surface condition suggests that the maximum temperature value in the direct heat treatment area was higher in some cases by the solidification temperature ($T_{s}$ = 1750 K). A comparative analysis of the surface was carried out using a Keyence VHX-S7000 series digital microscope for x30 magnification. Then, in order to determine surface quality parameters, a profile was made on a Form Talysurf 120 profilographometer by Taylor Hobson, obtaining measurements of waviness and roughness.

**Figure 10:**
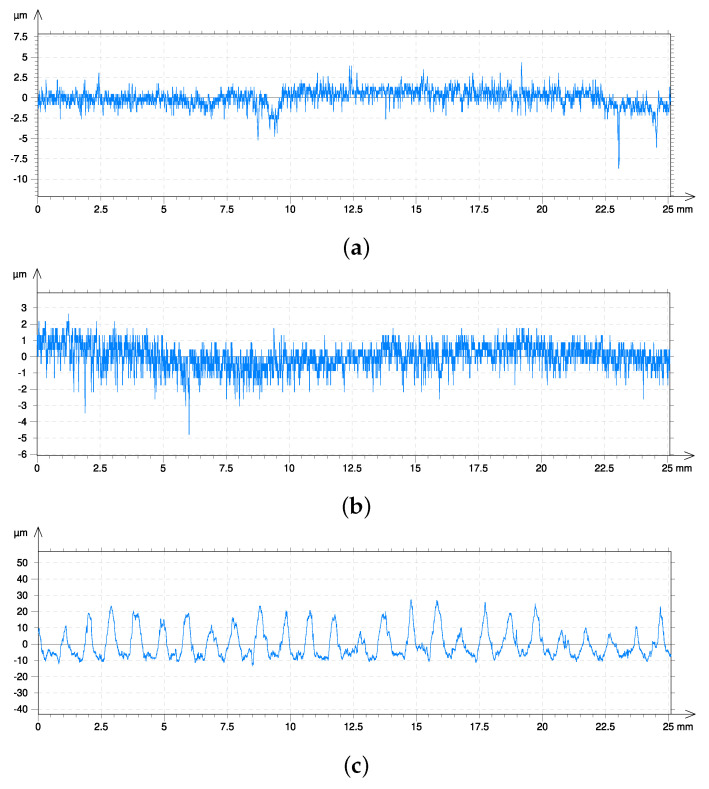

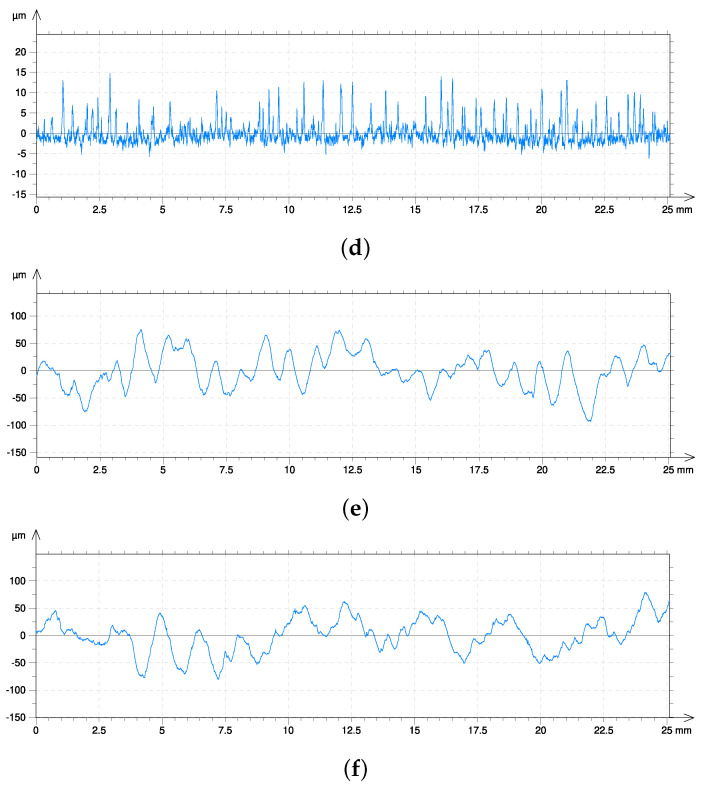
Surface condition of samples (profilograph (μm)) after the experiment, waviness + roughness: (**a**) *I* = 40 A, $\omega_{p}$ = 1 rot/s, (**b**) *I* = 40 A, $\omega_{p}$ = 2 rot/s, (**c**) *I* = 60 A, $\omega_{p}$ = 1 rot/s, (**d**) *I* = 60 A, $\omega_{p}$ = 2 rot/s, (**e**) *I* = 80 A, $\omega_{p}$ = 1 rot/s, (**f**) *I* = 80 A, $\omega_{p}$ = 2 rot/s.

The figures from the microscope show a fairly smooth surface only for the lowest current value (Figure 9a,b). The use of a rotational speed of 1 rot/s results in overheating on the path line of the heat source, however increasing the rotational speed (up to 2 rot/s) causes uniformity of the surface even for an intensity equal to 60 A (Figure 9d). On the other hand, the surface at the highest analysed arc current definitely shows quite deep remelting and the impact of corrosion processes (Figure 9e,f). The profilometer examination confirmed conclusions based on observations of the surface under the microscope. Due to editing limitations, it was decided to present the waviness and roughness as a sum on one diagram for each heating case (Figure 10).

Between 40 A and 60 A, a noticeable more than 10 times increase in waviness and between 60 A and 80 A increase in waviness of more than 300%. We also found more than a 4-times increase in roughness between 40 A and 60 A. In contrast, roughness does not increase significantly between 60 A and 80 A, and an increase at the level of 20% (Figure 10).

The number of rotations for 40 A does not significantly affect the waviness of the surface, but it has a significant effect for higher arc currents (Figure 10a,b). The level of waviness for 60 A is slightly greater than the waviness up to 40 A; however, the roughness is definitely greater with 60 A than with 40 A. The surface roughness between 60 A for 1 rotation and 2 rotations is in a comparable range with an indication of less for 2 rotations (Figure 10c,d). On the other hand, for 80 A, the waviness between single and double rotations is at a comparable level (Figure 10c,d). About the roughness for 80 A at 1 and 2 rotations, the same conclusions can be drawn as for 60 A (comparable level, with an indication of a lower value for 2 rotations).

## 6. Conclusions

Heating steel elements for heat treatment using the TIG method is an interesting alternative to classical heat treatment methods. Deliberate use of the disadvantage of the TIG method (shallow remelting) allows for achieving temperatures characteristic of the hardening process without a large modification of the surface. The condition of the surface, especially for higher current levels in the heat source suggests that remelting has occurred. This is noticeable in the figures showing the roughness, waviness (Figure 10) or in the images of the surface (Figure 9) in the form of remelted areas in the shape of a spiral path (60 A) and in the form of corrugations for 80 A. The numerical model in the presented form is not able to take this phenomenon into account.

The applied heat source model allows the correct modelling of temperature on a macroscopic scale for the heating process by looking at the obtained accuracy. In order to include the surface changes visible on both surface profiles and microscope figures, a different heat source model that takes into account phenomena at the microscopic scale and a much denser discretization of time and space would have to be used. To check the current numerical model, numerical simulations were also carried out for a mesh with 5-times smaller finite elements. This was to eliminate the effect of averaging over too large finite elements. However, such densification also did not show the possibility of remelting of the surface layer, which occurred and is visible in the experimental results. It should also be noted that some of the results confirming remelting may also relate to the process of surface degeneration due to oxidation and the use of gas shielding only for the heat source.

The presented work justifies the need to perform experimental studies calibrating parameters of numerical models, especially for values with a large range of changes. In fact, due to large approximation errors, it is impossible to set parameters for a model used commercially based on data for other process parameters. The characteristics of heat sources, the geometry of the workpiece and environmental conditions introduce large changes in the parameters of numerical models.

To summarize:

- The implemented numerical models allow the selection of such technological parameters as transition paths and current parameters of heat sources;
- The built research station as a demonstrator of heating technology with a TIG type source will allow further research in the field of different steel grades and geometries of axisymmetric elements;
- The necessity and effectiveness of the additional calibration of non-contact temperature measurement equipment has been demonstrated;
- The need for further work in the area of avoiding melting of the surface layer was demonstrated;
- Due to the selected parameters of the model, a satisfactory level of differences in relation to the presented experiment was obtained, which will allow the use of the calibrated model for the analysis of elements with variable cross-sections.

## Figures and Tables

**Table 1:** Summary of parameters of numerical simulations carried out.

Analysis	Rotation	Current	Voltage	Power	Radius of the
No.	(rot/s)	(A)	(V)	(W)	Heat Source (m)
1	1	40	9.5	266	0.0018
2	2				
3	1	60	9.8	411.6	0.00199
4	2				
5	1	80	10.7	599.2	0.00216
6	2				

**Table 2:** Difference of the results of the experiment and the numerical model in percentage (%).

Time (s)	Analysis No.					
	1	2	3	4	5	6
15	−4.49	3.25	−0.75	3.03	−3.77	−6.07
30	−1.56	6.21	−5.28	−5.11	2.54	0.27
45	0.28	3.39	−5.95	−5.51	4.69	2.26
60	0.39	1.06	−5.22	−5.28	5.63	3.52
90	0.35	−1.08	−4.28	−5.45	4.44	3.52

## Data Availability

Not applicable.
